# Supplementary material for: Differential allelic representation (DAR) identifies candidate eQTLs and improves transcriptome analysis
Source: bioRxiv. 2023 Mar 21:2023.03.02.530865. Preprint. [Version 3] doi: 10.1101/2023.03.02.530865 (PMC10028786; doi:10.1101/2023.03.02.530865)
Supplement: Supplement 1 [file NIHPP2023.03.02.530865v3-supplement-1.pdf]

bioRxiv preprint doi: https://doi.org/10.1101/2023.03.02.530865; this version posted March 22, 2023. The copyright holder for this preprint (which was not certified by peer review) is the author/funder, who has granted bioRxiv a license to display the preprint in perpetuity. It is made available under aCC-BY 4.0 International license.

SUPPLEMENTARY INFORMATION

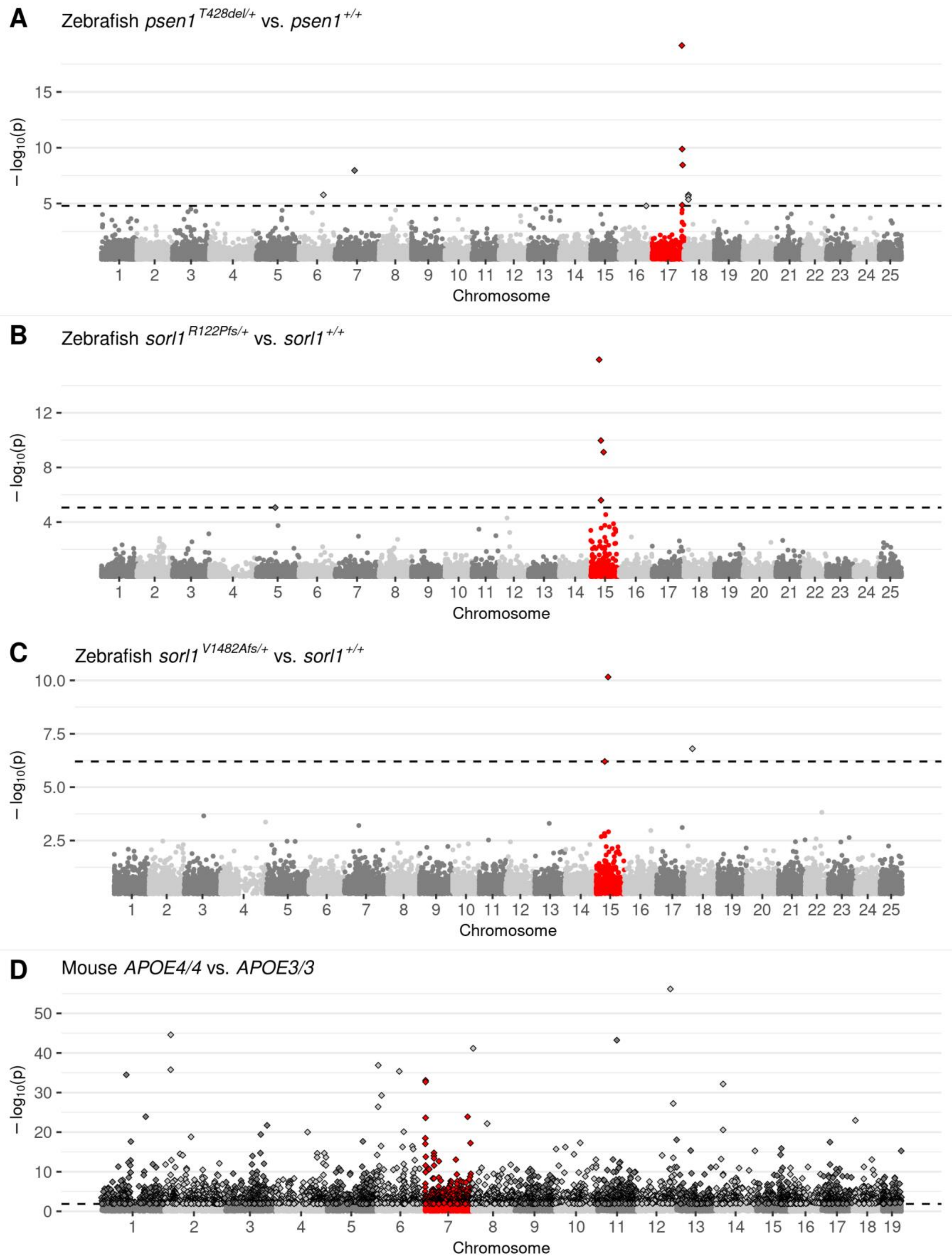

Supplementary Figure 1. Manhattan plots highlighting CC-DEG from differential expression testing in heterozygous vs. wild-type zebrafish siblings (6-month-old brains) and between homozygous mouse mutants (3-month-old male cortices). **A)** zebrafish EOfAD-like *psen1*$^{T428del/+}$ vs. *psen1*$^{+/+}$ **B)** zebrafish EOfAD-like *sorl1*$^{R122Pfs/+}$ vs. *sorl1*$^{+/+}$ **C)** zebrafish EOfAD-like *sorl1*$^{V1482Afs/+}$ vs. *sorl1*$^{+/+}$ **D)** mouse *APOE4/4* vs. *APOE3/3*. Non-random accumulation of DEGs was tested using a Bonferroni-adjusted Fisher's exact test *p*-value for

bioRxiv preprint doi: https://doi.org/10.1101/2023.03.02.530865; this version posted March 22, 2023. The copyright holder for this preprint (which was not certified by peer review) is the author/funder, who has granted bioRxiv a license to display the preprint in perpetuity. It is made available under aCC-BY 4.0 International license.

enrichment of DE genes on the mutant chromosome, A) *psen1*$^{T428del/+}$ vs. *psen1*$^{+/+}$: *p* = 8.63e-3, B) *sorl1*$^{R122Pfs/+}$ vs. *sorl1*$^{+/+}$: *p* = 1.92e-4, C) *sorl1*$^{V1482Afs/+}$ vs. *sorl1*$^{+/+}$: *p* = 9.24e-2, D) *APOE4/4* vs. *APOE3/3*: *p* = 1.00. Genes are plotted along the x-axis based on their chromosomal position in alternating shades of grey for visual distinction between chromosomes. Genes on the chromosome containing the mutation are highlighted in red. The raw *p*-values are plotted along the y-axis at the $-\log_{10}$ scale such that the most significant genes exist at the top of the plot. The cut-off for gene differential expression (FDR-adjusted *p*-value < 0.05) is indicated by a dashed horizontal line. Genes classified as differentially expressed under this criterion are represented as diamonds with a black outline.

bioRxiv preprint doi: https://doi.org/10.1101/2023.03.02.530865; this version posted March 22, 2023. The copyright holder for this preprint (which was not certified by peer review) is the author/funder, who has granted bioRxiv a license to display the preprint in perpetuity. It is made available under aCC-BY 4.0 International license.

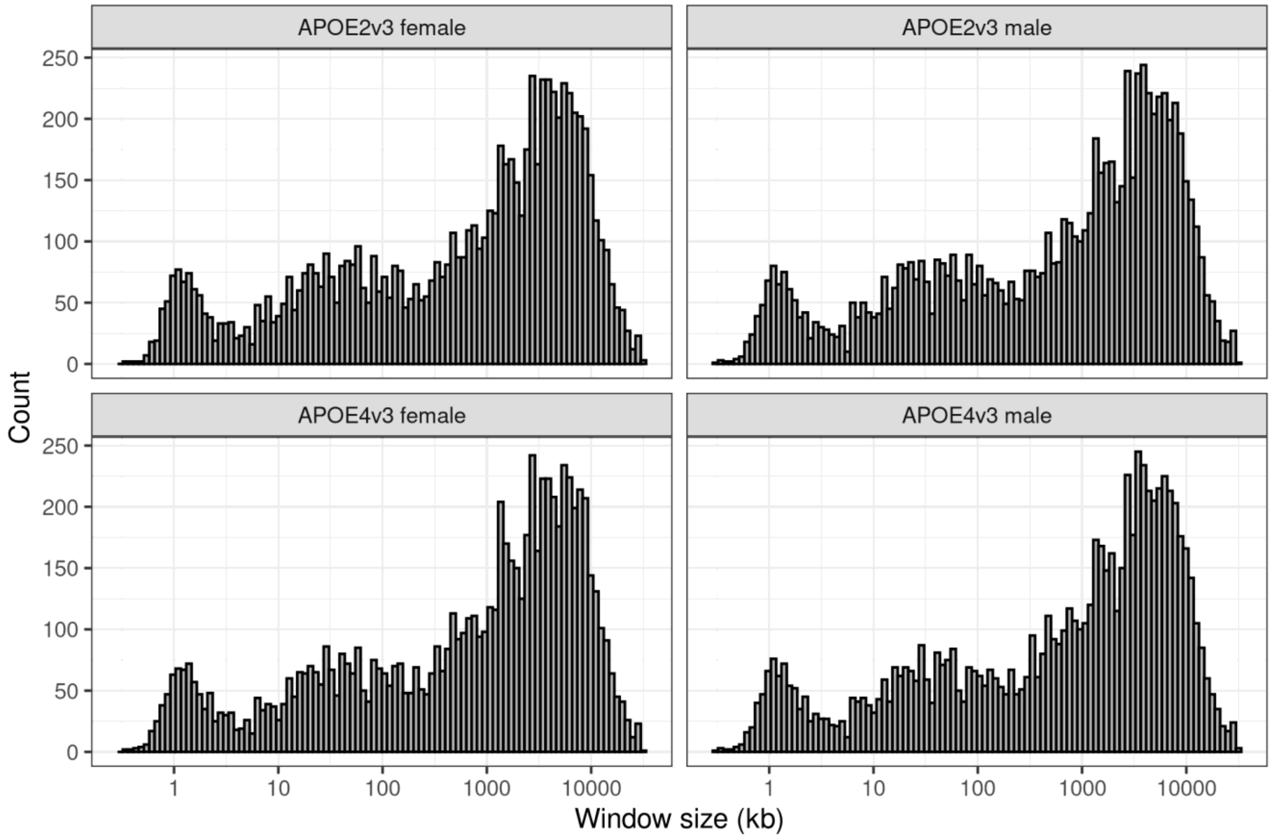

Supplementary Figure 2. Elastic window sizes of the mouse APOE dataset summarised in 100 bins of equal size. Window sizes are determined based on the genomic distance between 11 subsequent SNPs.

bioRxiv preprint doi: https://doi.org/10.1101/2023.03.02.530865; this version posted March 22, 2023. The copyright holder for this preprint (which was not certified by peer review) is the author/funder, who has granted bioRxiv a license to display the preprint in perpetuity. It is made available under aCC-BY 4.0 International license.

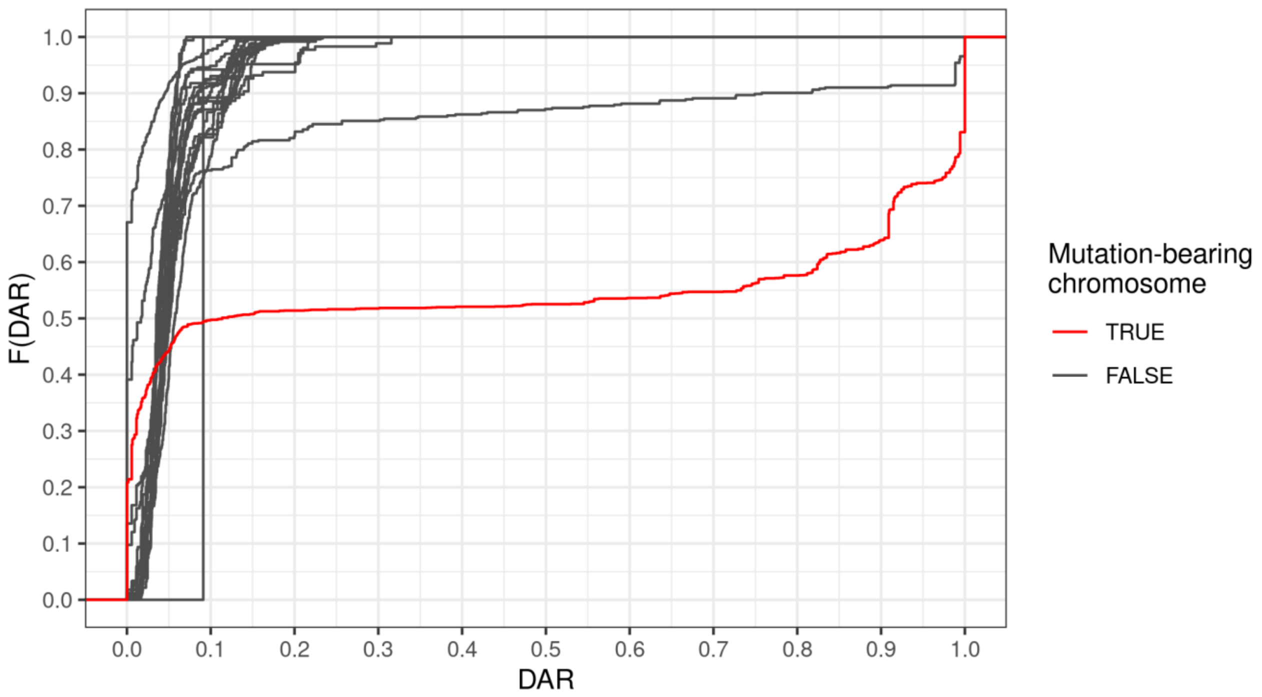

Supplementary Figure 3. Cumulative distribution of DAR by chromosome between male *APOE2*/*2* and *APOE3*/*3* mouse cortices (3 months). The mutant chromosome exhibits the most regions of high DAR.

bioRxiv preprint doi: https://doi.org/10.1101/2023.03.02.530865; this version posted March 22, 2023. The copyright holder for this preprint (which was not certified by peer review) is the author/funder, who has granted bioRxiv a license to display the preprint in perpetuity. It is made available under aCC-BY 4.0 International license.

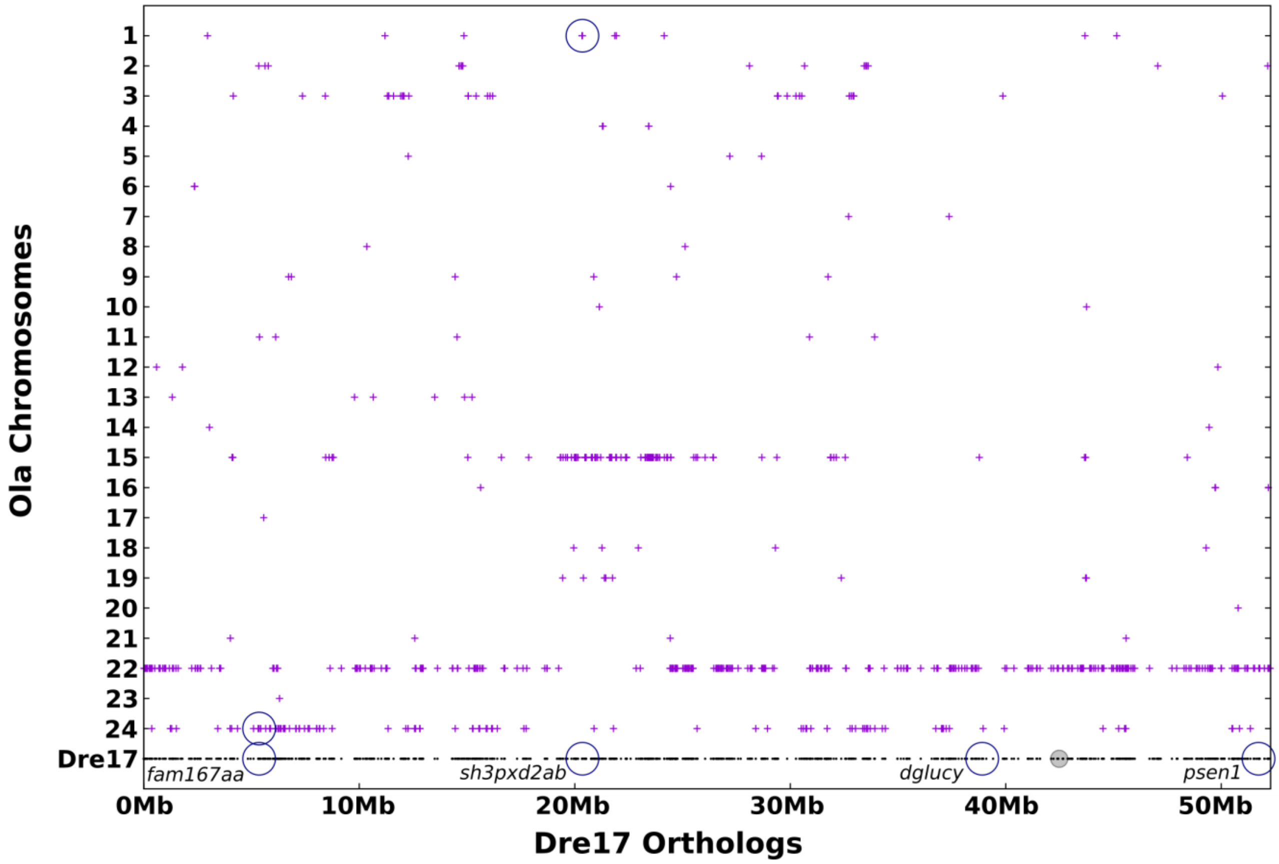

Supplementary Figure 4. Dotplot displaying the chromosomal location of medaka (Ola) genes predicted by the Synteny Database (32) to be orthologues of zebrafish (Dre) Chromosome 17 DE genes from the fAI-relevant, *psen1*$^{W233fs/+}$ vs. *psen1*$^{+/+}$ comparison. Genes are plotted along the x-axis based on their position on Chromosome 17 in zebrafish, while the y-axis indicates the chromosome they are located on in medaka. The four DE genes predicted to have orthologues are circled in blue. The two genes without a secondary blue circle plotted along the y-axis (*dglucy* and *psen1*), were predicted by the Synteny Database to exist on alternate scaffolds of medaka Ensembl version 71, which are not plotted.

bioRxiv preprint doi: https://doi.org/10.1101/2023.03.02.530865; this version posted March 22, 2023. The copyright holder for this preprint (which was not certified by peer review) is the author/funder, who has granted bioRxiv a license to display the preprint in perpetuity. It is made available under aCC-BY 4.0 International license.

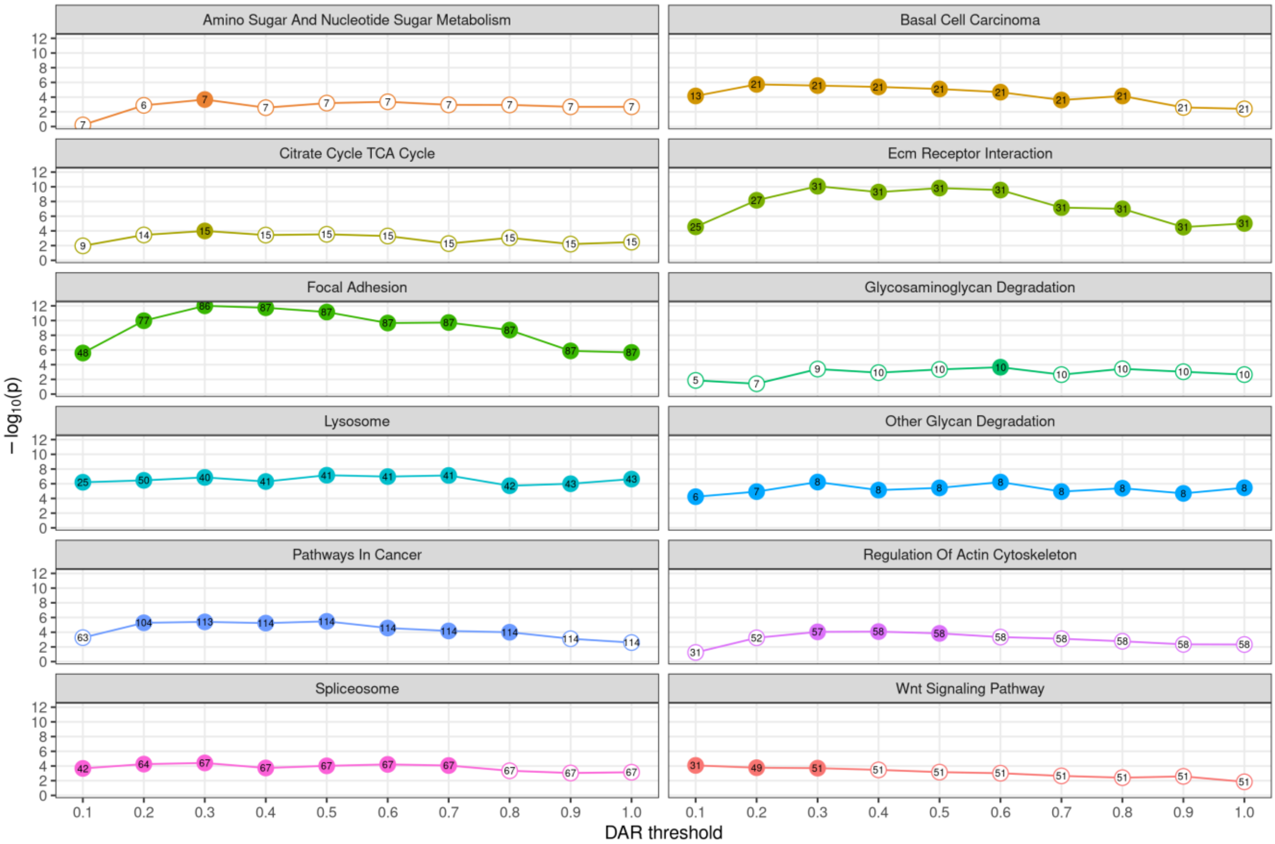

Supplementary Figure 5. The effects of gene exclusion by DAR threshold on KEGG gene set *p*-values from GSEA testing. The gene sets displayed are those that achieved significance for at least one DAR threshold value. *p*-values are plotted on a $-\log_{10}$ scale along the y-axis such that the most significant results exist at the top of each graph. Each position along the x-axis represents a different DAR gene exclusion threshold. Dots on the graph filled with colour indicate that the gene set was classified as significantly enriched (FDR-adjusted *p*-value < 0.05). The number inside a dot corresponds to the number of leading-edge genes that contributed to the respective gene set's enrichment score.

bioRxiv preprint doi: https://doi.org/10.1101/2023.03.02.530865; this version posted March 22, 2023. The copyright holder for this preprint (which was not certified by peer review) is the author/funder, who has granted bioRxiv a license to display the preprint in perpetuity. It is made available under aCC-BY 4.0 International license.

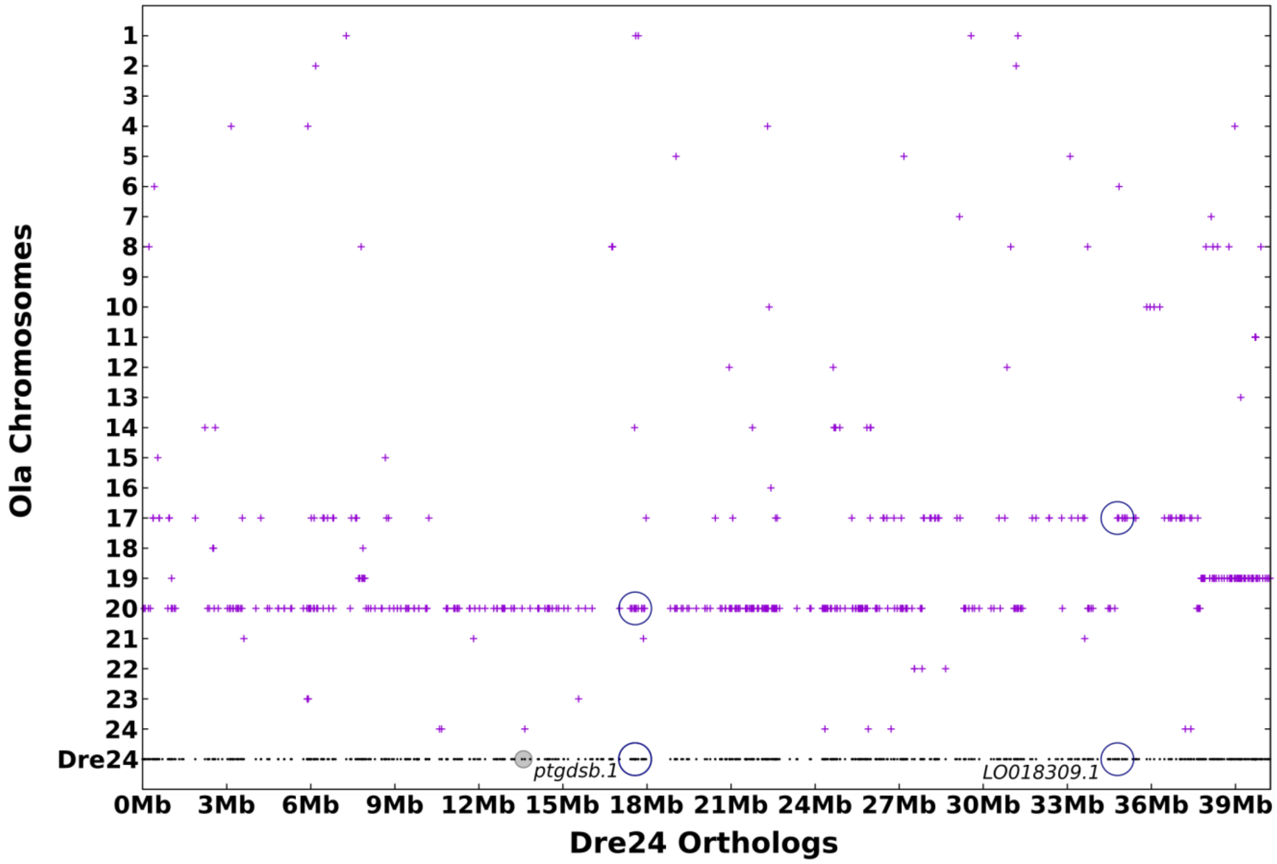

Supplementary Figure 6. Dotplot displaying the chromosomal location of medaka (Ola) genes predicted by the Synteny Database (32) to be orthologues of zebrafish (Dre) Chromosome 24 DE genes from the *naglu*$^{A603Efs/A603Efs}$ vs. *naglu*$^{+/+}$ 7 dpf sibling larval RNA-seq dataset comparison at a DAR threshold of 0.3. Genes are plotted along the x-axis based on their position on Chromosome 24 in zebrafish, while the y-axis indicates the chromosome they are located on in medaka. The two DE genes predicted to have orthologues are circled in blue.

bioRxiv preprint doi: https://doi.org/10.1101/2023.03.02.530865; this version posted March 22, 2023. The copyright holder for this preprint (which was not certified by peer review) is the author/funder, who has granted bioRxiv a license to display the preprint in perpetuity. It is made available under aCC-BY 4.0 International license.

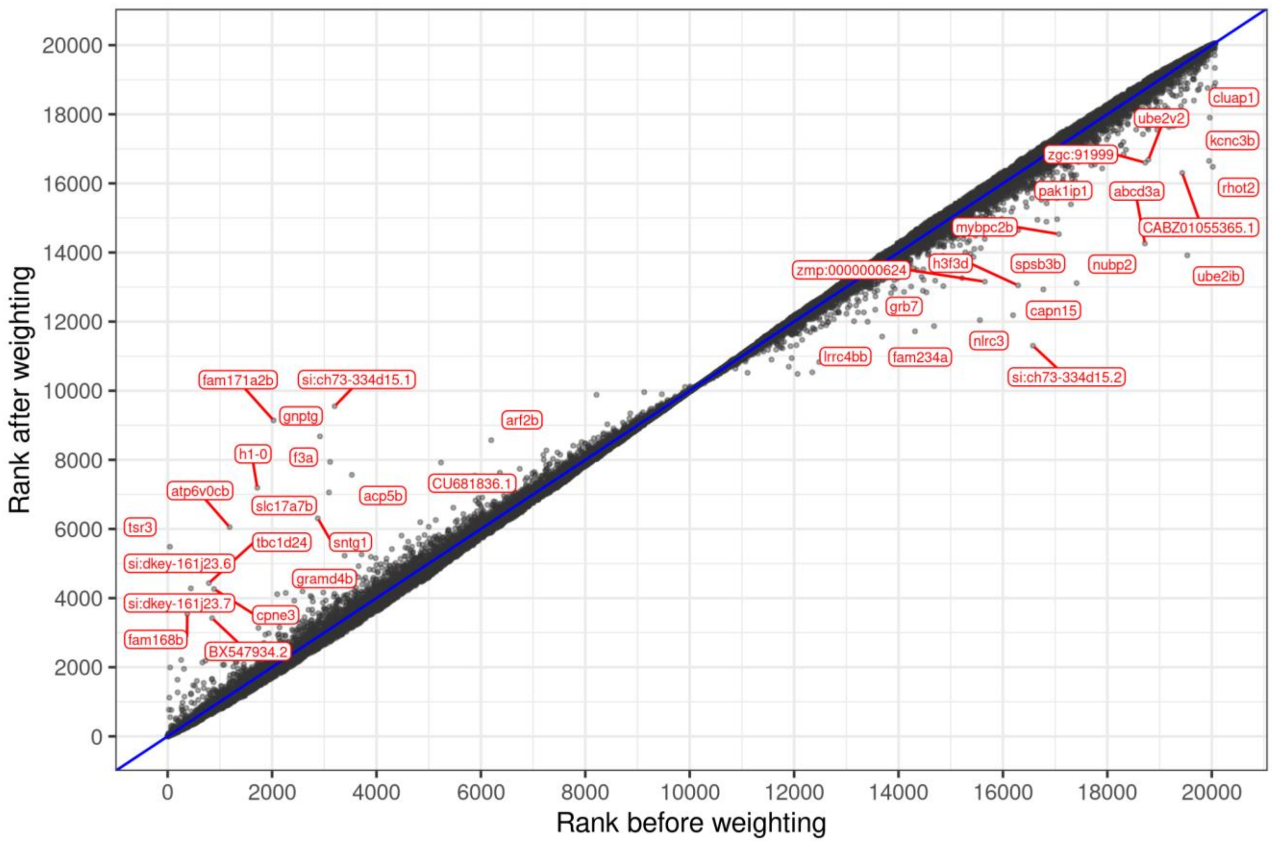

Supplementary Figure 7. The effects of using DAR to weight gene-level rankings in the MPS-IIIB-relevant transcriptome comparison of *naglu*$^{A603Efs/A603Efs}$ vs. *naglu*$^{+/+}$ 7 dpf sibling larvae. Genes that are plotted close to the blue diagonal line are least impacted by the weighting method. Genes that were most substantially affected (rank change > 2000) are labelled in red.

bioRxiv preprint doi: https://doi.org/10.1101/2023.03.02.530865; this version posted March 22, 2023. The copyright holder for this preprint (which was not certified by peer review) is the author/funder, who has granted bioRxiv a license to display the preprint in perpetuity. It is made available under aCC-BY 4.0 International license.

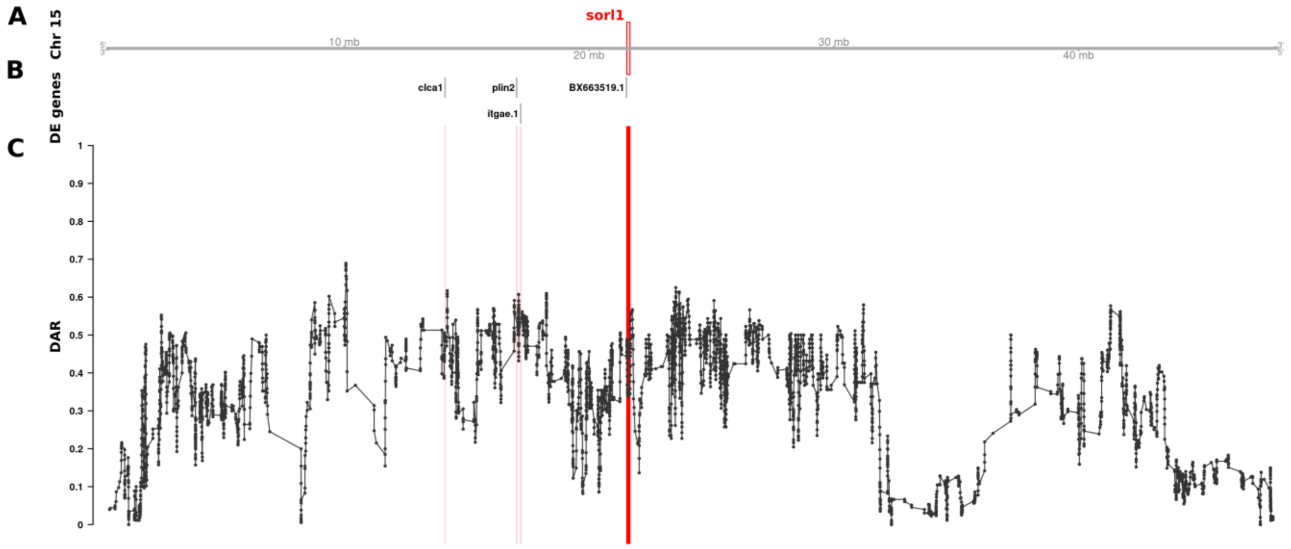

Supplementary Figure 8. The relationship between DAR and DE genes along Chromosome 15 in the comparison of *sorl1*$^{R122Pfs/+}$ vs. *sorl1*$^{+/+}$ sibling zebrafish brains. The plot contains four sets of information represented by separate tracks. Track A represents the axis of Chromosome 15. The position of the *sorl1* gene is marked and labelled in bold red. Track B displays differentially expressed genes according to their positions along the chromosome. Track C shows the trend in DAR as a connected scatterplot with each point representing the DAR value at a single nucleotide variant position (elastic sliding window, n = 11 variants). Positions of the DE genes shown in track B are indicated by light red lines.

bioRxiv preprint doi: https://doi.org/10.1101/2023.03.02.530865; this version posted March 22, 2023. The copyright holder for this preprint (which was not certified by peer review) is the author/funder, who has granted bioRxiv a license to display the preprint in perpetuity. It is made available under aCC-BY 4.0 International license.

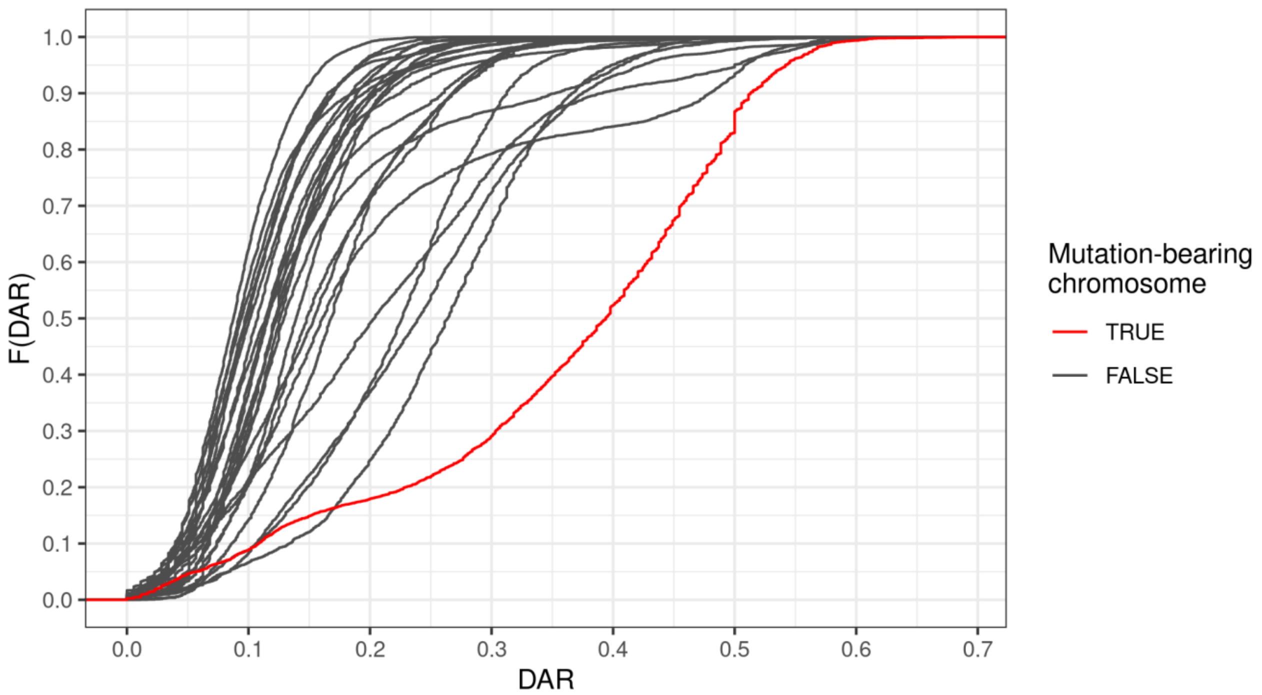

Supplementary Figure 9. Cumulative distribution of DAR by chromosome between *sorl1*$^{R122Pfs/+}$ and *sorl1*$^{+/+}$ sibling zebrafish. The mutant chromosome exhibits the most regions of high DAR.

bioRxiv preprint doi: https://doi.org/10.1101/2023.03.02.530865; this version posted March 22, 2023. The copyright holder for this preprint (which was not certified by peer review) is the author/funder, who has granted bioRxiv a license to display the preprint in perpetuity. It is made available under aCC-BY 4.0 International license.

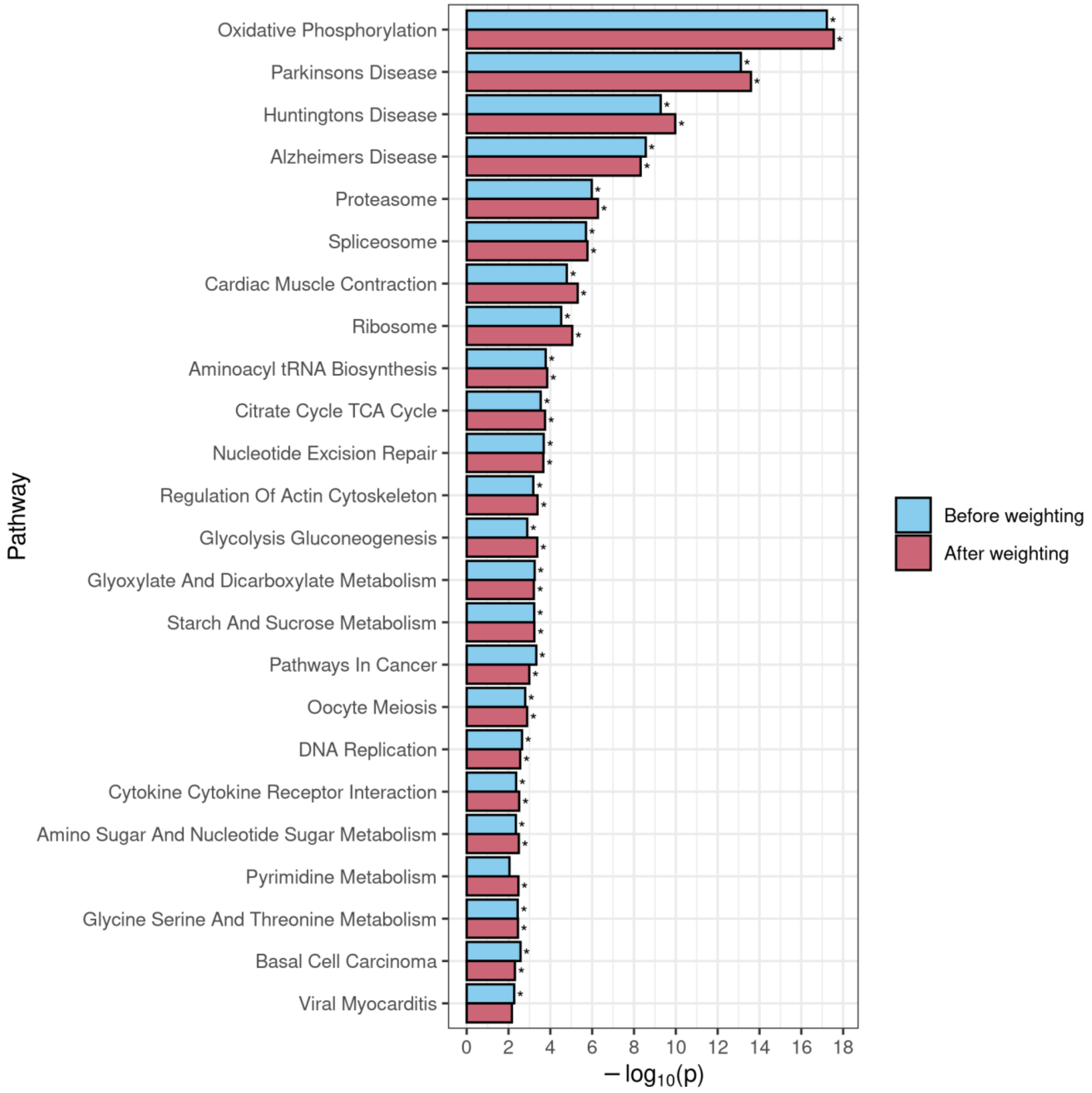

Supplementary Figure 10. Comparison of GSEA results for KEGG gene sets that achieved significance before and/or after using DAR to weight the gene-level ranking statistic in the *sorl1*$^{R122Pfs/+}$ vs. *sorl1*$^{+/+}$ siblings dataset. An asterisk is used to denote that the pathway was determined to be significantly enriched.
